# Fully Covered Self‐Expandable Metal Stent Placement for Management of a Perforated Duodenal Ulcer With Persistent Leak in a Decompensated Cirrhotic

**DOI:** 10.1155/crgm/1608984

**Published:** 2025-12-22

**Authors:** James Vu, Robert L. Pecha, Andrew W. Yen, Vikrant Rachakonda

**Affiliations:** ^1^ Department of Internal Medicine, UC Davis Medical Center, Sacramento, California, USA, ucdavis.edu; ^2^ Division of Gastroenterology and Hepatology, UC Davis Medical Center, Sacramento, California, USA, ucdavis.edu; ^3^ Division of Gastroenterology, Sacramento VA Medical Center, Veterans Affairs Northern California Health Care System (VANCHCS), Sacramento, California, USA

## Abstract

Patients with cirrhosis have a high prevalence of peptic ulcer disease, which places them at increased risk for complications such as perforation. We report the case of a patient with decompensated cirrhosis who developed a duodenal ulcer perforation following an upper endoscopy for variceal screening. Due to poor surgical candidacy, he was managed conservatively through antibiotics, percutaneous drains, and endoscopic placement of a fully covered self‐expandable metal stent (fcSEMS). With no established guidelines on managing duodenal perforations in cirrhotic patients, this case demonstrates a successful outcome with a nonoperative approach that can be considered when definitive surgical intervention is not feasible.

## 1. Introduction

Duodenal ulcer perforation is a rare but life‐threatening condition, with mortality ranging from 8% to 25% [1]. Previous studies have noted that in addition to higher bleeding risk, the prevalence of peptic ulcer disease (PUD) is higher in cirrhotic patients relative to the general population [2, 3]. While the management of duodenal perforation traditionally involves surgery, surgical complications and mortality are higher in cirrhotic patients [4, 5]. The World Society of Emergency Surgery (WSES) guidelines from 2020 state that while not the standard of care, there may be cases of duodenal perforation in nonoperative candidates in which endoscopic stent placement and percutaneous or laparoscopic drain placement can be an alternative to surgery [4]. Here, we present a case of duodenal perforation in a patient with decompensated cirrhosis that was successfully managed through percutaneous drains and placement of a fully covered self‐expandable metal stent (fcSEMS).

## 2. Case Report

**Figure 1:**
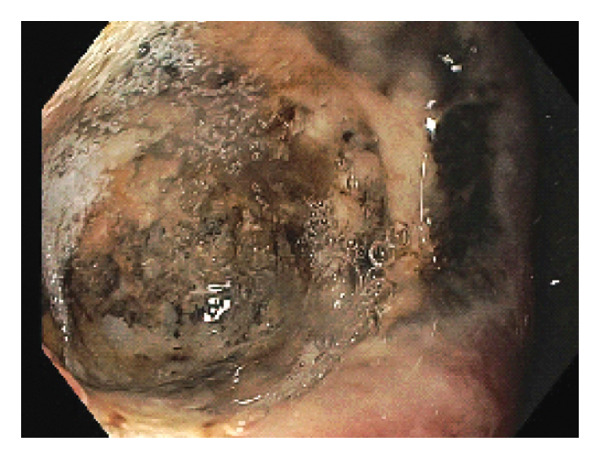
Initial endoscopic examination revealing a > 2 cm nonbleeding, deep cratered ulcer in the duodenal bulb.

A 69‐year‐old male with alcohol‐related cirrhosis complicated by hepatic encephalopathy and diuretic‐refractory ascites underwent esophagogastroduodenoscopy (EGD) for variceal screening. He had no prior history of *H. pylori* infection or NSAID use. The EGD was notable for two small columns of varices in the distal esophagus, multiple small erosions in the gastric antrum, and mild portal hypertensive gastropathy. In the duodenal bulb, an approximately 2 cm nonbleeding, cratered ulcer with heaped‐up edges was identified along the anterior wall, extending into the duodenal sweep (Figure 1). No immediate periprocedural complications were noted, and the patient did not report any new symptoms during his initial monitoring period.

The next morning, the patient developed severe abdominal pain and altered mental status. He was afebrile but tachycardic. Given his acute onset of symptoms, a paracentesis was performed, and ascitic fluid analysis revealed 19,000 WBC with 90% neutrophils, consistent with peritonitis. An abdominal CT showed free peritoneal air concerning for duodenal bulb perforation (Figures 2(a) and 2(b)). Given his history of Child–Pugh Class C cirrhosis and a MELD 3.0 score of 21, he was deemed a prohibitively poor surgical candidate. He was therefore managed conservatively with bowel rest, parenteral nutrition, broad‐spectrum antibiotics, and high‐dose intravenous proton pump inhibitor therapy.

Figure 2Computed tomography of the abdomen showing: (a) intraabdominal free air underneath the hemidiaphragm and (b) mildly increased density of fluid in same space which could represent mixing of leaked oral contrast.

**Figure:**
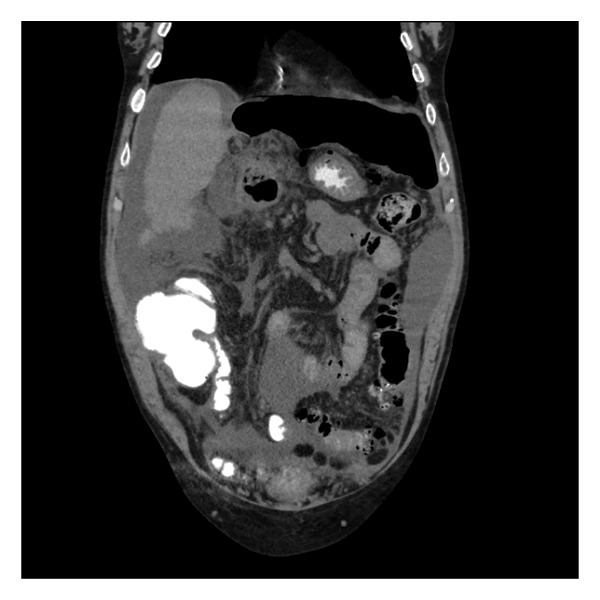
(a)

**Figure:**
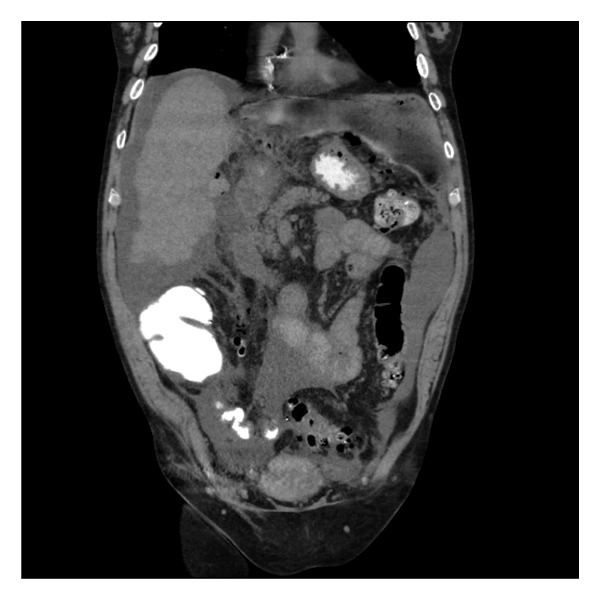
(b)

**Figure 3:**
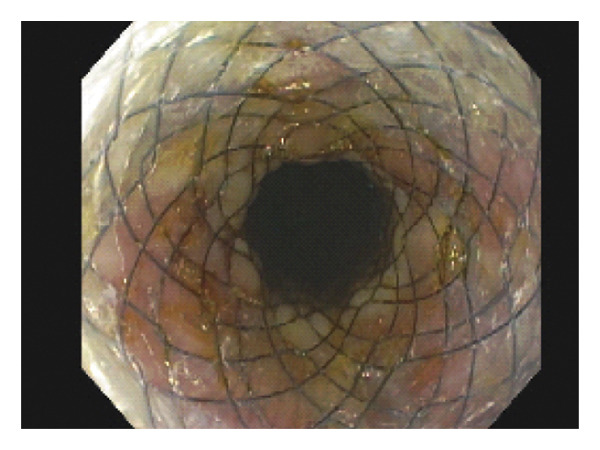
Follow‐up endoscopy with visualization of fully expanded fcSEMS across the site of duodenal ulcer perforation.

Over the next week, the patient had persistent tachycardia and leukocytosis. An abdominal X‐ray noted a left upper quadrant fluid collection, concerning for a loculated abscess. Interventional radiology was consulted and placed two percutaneous drains in the superior and inferior left hemiabdomen, adjacent to the abscess and the perforated duodenal ulcer. Given the concern for a persistent leak, the decision was made to proceed with endoscopic management. On repeat EGD, a 9 mm ulcer was found along the proximal anterior duodenal bulb just distal to the pylorus, with improved size and surrounding inflammation compared to the appearance on initial EGD. Initially, given the size of the defect, closure was attempted with through‐the‐scope (TTS) endoclips; however, this approach failed due to unfavorable positioning and surrounding fibrosis. Primary closure with an over‐the‐scope clip or endoscopic suturing was felt to have a low likelihood of technical success given the difficult location and approach to the perforation. A TTS 20 mm × 80 mm fcSEMS esophageal stent (Olympus Hanarostent) was then deployed under endoscopic and fluoroscopic visualization. A fully covered esophageal stent was selected for off‐label use in this case, given the limited availability of fully covered gastroduodenal stents in the United States. The proximal edge of the stent with the flange was positioned in the stomach proximal to the pylorus. Two TTS clips were placed on the lasso loop in the antrum to further secure the stent in position. The stent traversed the duodenal ulcer, and its distal edge was positioned in D2 (Figure 3).

Following the procedure, the patient had clinical improvement with resolution of his leukocytosis while remaining on a prolonged course of IV antibiotics with percutaneous drains in place. He was kept strictly *nil per os*, and total parenteral nutrition (TPN) was initiated, which he remained on for 3 weeks. Initiation of enteral nutrition was postponed, avoiding the risk of peritoneal contamination based on an early poststent oral contrast upper gastrointestinal radiographic study suggesting a possible small, persistent leak. Although a subsequent CT scan with oral contrast did not confirm a leak, based on the patient’s prohibitive surgical risk and in an abundance of caution, parenteral nutrition was initially favored. However, due to concerns for volume overload and worsening ascites, he was gradually transitioned to a full liquid diet, which he tolerated well, and TPN was subsequently discontinued. A follow‐up CT abdomen with oral contrast did not demonstrate evidence of abscess or contrast extravasation from the site of duodenal ulcer.

**Figure 4:**
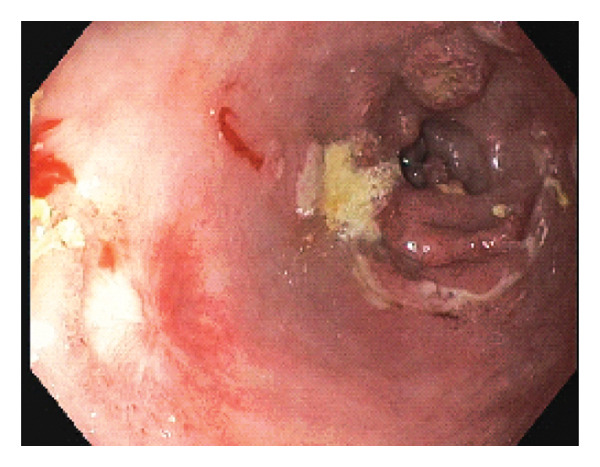
The appearance of the mucosa following stent removal, revealing a healed prior ulcer.

Given that there was no evidence of a leak on imaging, the patient underwent EGD 6 weeks later for stent removal and the prior perforated duodenal ulcer site appeared well‐healed and without high‐risk stigmata (Figure 4). His percutaneous drains were without output and were successfully removed 2 days after stent removal. The patient remained clinically stable and was discharged 1 week later to a skilled nursing facility for prolonged physical rehabilitation. He remained in stable condition during his 3‐month stay and was later discharged home. At a follow‐up clinic visit, he remained asymptomatic without abdominal pain, fever, or leukocytosis.

## 3. Discussion

The prevalence of PUD in cirrhotic patients is higher relative to the general population [3]. While traditional factors such as *H. pylori* and NSAID use play a role, the influence of cirrhosis on mucosal blood flow, defense, and wound healing may also contribute [6, 7]. Ulcer perforation, though a less common complication of PUD, carries the highest mortality rate, with gastroduodenal perforations in cirrhotic patients associated with significantly worse outcomes (mortality rate of 62.5% versus 16.6% in noncirrhotic patients) [8, 9]. Management of perforations in these patients poses significant challenges, as their prognosis is significantly worse due to the limited management options available and the high risk of mortality with surgery in this population [10, 11].

To the best of our knowledge, this is the first reported case demonstrating the use of an fcSEMS in delayed (> 1 week out) management of a duodenal perforation with persistent leak after failure of conservative therapies in a patient with decompensated cirrhosis. The cases describing the use of fcSEMS for duodenal perforations are limited to early endoscopic management in noncirrhotic patients or those with postoperative leakage after initial traditional surgical closure; however, these cases highlight a similar approach with a favorable outcome [12–15].

Currently, there is no literature regarding the endoscopic management of perforations in cirrhotic patients. However, the 2021 AGA Clinical Practice Update on Endoscopic Management of Perforations in the Gastrointestinal Tract provides general principles when treating duodenal perforations. Generally, early endoscopic closure following perforation should be attempted with TTS clips, over‐the‐scope clips, band ligation, or endoloops [16]. Early closure is beneficial, as the perforation margins are less inflamed, increasing the likelihood of success when utilizing clips [17]. If early closure is not feasible, the off‐label use of fully covered esophageal stents may be reasonable to seal perforations, though this approach carries a risk of stent migration [18]. However, this risk can be mitigated if the proximal end is secured by anchoring techniques such as endoscopic suturing, as demonstrated in our case [16]. Cirrhotic patients are unique—they have impaired wound healing, so primary closure of luminal defects is preferred [10]. As demonstrated here, this approach offers the potential for reduced morbidity through nonoperative management, particularly in patients at risk for prolonged or complicated recovery. This report highlights the technical and clinical success of endoscopic management of a duodenal perforation with an fcSEMS in a patient with decompensated cirrhosis when primary closure is not feasible and conservative measures fail.

Management of perforations in cirrhotic patients or those who are nonsurgical candidates remains a significant clinical challenge; further work is needed to establish evidence‐based recommendations for endoscopic and surgical management, but fcSEMS should be part of the endoscopist’s toolkit.

## Consent

Informed patient consent was obtained for this case report publication.

## Disclosure

The contents reported/presented within do not necessarily represent the views of the Department of Veterans Affairs or the United States Government.

## Conflicts of Interest

The authors declare no conflicts of interest.

## Funding

No funding was received for this manuscript.

## Data Availability

Data sharing is not applicable to this article as no datasets were generated or analyzed during the current study.
